# *In vitro* Bioassay and *In silico* Pharmacokinetic Characteristics of *Xanthium strumarium* Plant Extract as Possible Acaricidal Agent

**DOI:** 10.2174/0113816128317849241108064144

**Published:** 2024-12-17

**Authors:** Nabi Amin, Chia-Hung Wu, Nosheen Malak, Afshan Khan, Shakir Ullah, Imtiaz Ahmad, Muazzam Ali Khan, Muhammad Naveed, Zakir Ullah, Saira Naz, Adil Khan, Chien-Chin Chen

**Affiliations:** 1 Department of Botany and Zoology, Bacha Khan University, Peshawar, Charsadda 24420, Khyber Pakhtunkhwa, Pakistan;; 2 Division of General Surgery, Department of Surgery, Ditmanson Medical Foundation Chia-Yi Christian Hospital, Chiayi 600, Taiwan;; 3 Department of Zoology, Abdul Wali Khan University Mardan, Mardan 23200, Pakistan;; 4 Department of Chemistry, Bacha Khan University, Peshawar, Charsadda 24420, Khyber Pakhtunkhwa, Pakistan;; 5 Department of Pathology, Ditmanson Medical Foundation Chia-Yi Christian Hospital, Chiayi 600, Taiwan;; 6 Department of Cosmetic Science, Chia Nan University of Pharmacy and Science, Tainan 717, Taiwan;; 7 Rong Hsing Research Center for Translational Medicine, National Chung Hsing University, Taichung 402, Taiwan;; 8 Department of Biotechnology and Bioindustry Sciences, College of Bioscience and Biotechnology, National Cheng Kung University, Tainan 701, Taiwan

**Keywords:** *Xanthium strumarium*, larval packet test (LPT), adult immersion test (AIT), molecular docking, pharmacokinetic characteristics, acaricidal agent

## Abstract

**Background:**

Effective management strategies against tick infestations are necessary because tick-borne diseases represent serious hazards to the health of humans and animals worldwide. The aim of this study was to examine the larvicidal and ovicidal properties of *Xanthium strumarium* extract against a notorious tick species, *Rhipicephalus microplus*.

**Methodology:**

The maceration method was used to prepare the ethanolic extract of *X. strumarium.* The extract was then used in an adult immersion test (AIT) and larval packet test (LPT) to asses the plant's toxicity. To elucidate the mode of action, molecular modeling and docking studies were conducted. ADMET analysis was then carried out to find out the drug-likeness profiles of the plant phytochemicals.

**Results:**

Significant death rates and egg inhibition were found at different extract doses using the larval packet test (LPT) and adult immersion test (AIT). A concentration-dependent impact was observed at a concentration of 40 mg/mL, which resulted in the maximum larval mortality (92 ± 2.646) and egg inhibition (77.057 ± 2.186). Additionally, the potency of the extract against *R. microplus* was determined by calculating its fatal concentrations (LC_50_, LC_90_, and LC_99_). A three-dimensional model of the *R. microplus* octopamine receptor was created, and docking studies showed that the receptor and possible ligands, most notably Xanthatin and Xanthosin, interacted well. The potential of compounds as tick control agents was highlighted by their pharmacokinetic characteristics and toxicity profiles, as revealed by drug-likeness and ADMET studies. Molecular dynamic simulations further demonstrated the stability of the protein-ligand complex, indicating the consistent association between the ligand and the target protein.

**Conclusion:**

Overall, this study provides valuable insights into the potential use of *X. strumarium* extract and its compounds as larvicidal and ovicidal agents against *R. microplus*, paving the way for further research on tick control strategies.

## 1 INTRODUCTION

Ticks, external blood-feeding parasites found on vertebrate animals, including birds, mammals, and reptiles, serve as carriers of infectious agents, posing threats to humans, wildlife, and the cattle industry globally [1]. This dual concern in veterinary and public health reflects the potential adverse impact on animal productivity and well-being, either directly through bites or indirectly by transmitting pathogens, such as bacteria, rickettsia, viruses, and protozoa [2, 3]. The estimated economic impact of ticks and related diseases ranges from 13.9 to 18.7 billion USD, highlighting the widespread and substantial consequences [4]. Specifically, the cattle industry grapples with the formidable challenge posed by *Rhipicephalus microplus*, a widely distributed tick species with an annual economic impact of $US 25-30 billion on the global livestock sector [5, 6]. Recognized for its role in spreading infections like Babesiosis and Anaplasmosis, *R. microplus* significantly affects livestock, leading to the clinical condition known as “tick fever” and causing various symptoms, including anemia, weight loss, decreased milk production, and low leather quality [6-9].

Efforts to manage *R. microplus* involve the use of various synthetic acaricides, including organochlorides (OCs), organophosphates (OPs), amitraz, and synthetic pyrethroids (SPs) [10]. However, tick populations have increased as a result of the ongoing use of these acaricides, which exhibit resistance not only to acaricides but also to other medications [11, 12]. Genetic changes in ticks play a crucial role in the development of acaricide resistance, leading to alterations at the target site, enhanced acaricide metabolism, or reduced medication penetration through the outer protective layer of the tick [13, 14].

In addressing acaricide resistance across diverse tick species, researchers are exploring the potential of various plant essential oils and extracts as alternative solutions for tick management [15, 16]. The use of plant extracts not only has been associated with decreased environmental and food pollution but also contributes to slower rates of arthropod resistance development, offering reduced toxicity to both humans and animals [17]. A novel approach to tick management involves employing secondary metabolites, which can impact the outer layer development of the tick, inhibit its growth, maturation, and fertility, or influence its behavior, all while minimizing effects on non-target species.

The annual plant *Xanthium strumarium* L. belongs to the Asteraceae family and exhibits a diverse range of pharmacological properties, including effects on the neurological and digestive systems, analgesic and anti-inflammatory properties, antioxidant activity, hypoglycemic effects, anti-cancer properties, antibacterial and antifungal properties, anti-trypanosomal activity, and anti-tussive activity [18]. Numerous studies have documented the biocidal activity of essential oils against clinically significant infections [19, 20].

Octopamine, a versatile biogenic amine, serves crucial roles as a neurotransmitter, neurohormone, and neuromodulator in invertebrate systems, particularly influencing physiological processes, such as reproduction and oviposition [21, 22]. As an integral component of the tyraminergic/octopaminergic system, octopamine likely modulates neural circuits and hormonal pathways, notably in the regulation of egg-laying processes in female ticks [23]. Essential oils and their purified constituents, including eugenol, α-terpineol, and cinnamic alcohol, demonstrate neurotoxic and cytotoxic activities against arthropods, potentially through binding to the receptors for tyramine and octopamine, leading to lethal effects [24-26]. Given the need for efficient drug development methodologies, molecular docking emerges as a popular computational screening technique, predicting the structure and binding affinity of receptor-ligand complexes [27, 28].

The primary focus of this study was to conduct *in vitro* tests assessing the acaricidal effects of the essential oil derived from *X. strumarium* leaves. This study employed molecular docking to investigate the efficacy of *X. strumarium* in addressing tick infestations at different growth stages, encompassing larvae, nymphs, and adults. Additionally, *in silico* methods were utilized to identify plant-based compounds with anti-tick properties, offering a streamlined approach compared to labor-intensive laboratory screening.

## 2 MATERIALS AND METHODS

### 2.1 Plant Collection, Identification, and Extract Preparation

The plant parts selected for the study were collected from district Charsadda in Khyber Pakhtunkhwa, Pakistan (coordinates: 34.1682° N, 71.7504° E). Following collection, the plant materials underwent thorough cleaning and inspection for any damage. The leaves were subsequently identified as *X. strumarium* at the Department of Botany, Bacha Khan University, Charsadda herbarium. After identification, the leaves were air-dried for three weeks. Following a 22-day drying period, the leaves were powdered after being finely ground. Then, 50 g of stock solutions were made by dissolving the powdered leaves in 500 mL of 95% ethanol. The resulting mixture was incubated in a shaking incubator at 37°C and 250 rpm for 72 hours. Subsequently, it underwent a three-time filtration process and was evaporated at 48°C to obtain a thick solution, which was reduced to less than 5% of the original volume using a water bath. To assess the effectiveness of the extract, the stock solution was further diluted to concentrations of 25, 50, 75, and 100 mg/mL.

### 2.2 Collection and Identification of the Ticks

Following the guidelines set forth by the “World Association for the Advancement of Veterinary Parasitology” [29], adult *R. microplus* ticks were collected from sheep and cattle across multiple farms in district Buner, Khyber Pakhtunkhwa (coordinates: 34.1986° N, 72.0404° E). The collected ticks underwent a thorough cleaning process by rinsing them in distilled water and were subsequently identified using standard tick identification keys under a microscope [30]. A total of approximately 300 adult ticks were selected to be included in the study. Subsequently, the adult immersion test was conducted using these ticks, with the primary objective of assessing the acaricidal properties of the selected plant extract.

### 2.3 Bioassays against Tick Larvae and Adults

The effectiveness of the extract against the targeted tick species was assessed using the Adult Immersion Test (AIT) and Larval Packet Test (LPT), which were carried out in accordance with the previously published procedure by Ayub *et al*., in 2023. Seven groups were created on the *R. microplus* for use in LPT and AIT. Using the following formula, the percentage of egg inhibition (inhibition of oviposition, or % IO) was determined:


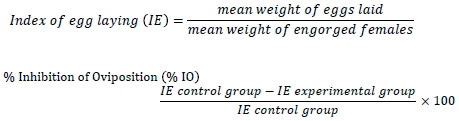


A control group was established using permethrin as the positive control and distilled water as the negative control to facilitate a comparison of the extract's results. All experiments were replicated three times on separate days, each with newly collected tick species.

### 2.4 *R. microplus* Octopamine Protein Sequence

The protein sequence of *R. microplus* octopamine was acquired by utilizing the accession number A7TZ09_RHIMP from the UniProt database (http://www.uniprot.org/) [31]. Through the (UniProt Knowledgebase tool) Uniprot, a freely accessible database, extensive information was obtained about protein sequences and their functions.

### 2.5 Validation of the Modelled Structures by Homology Modelling

Two homology modeling programs, namely Phyre2 [32] and the Swiss model [33], were utilized to generate the 3D structure of the proteins. The accuracy and quality of the modeled structure were assessed using evaluation tools, such as PROCHECK [34] and PROSA programs. Additionally, the ERRAT server was employed to examine the overall quality of the predicted structure. Among the generated structures, the one that exhibited better validation and acceptability was chosen for further investigation.

### 2.6 Prediction of Active Site

The process of predicting binding sites for the octopamine protein of the cattle tick involved the CASTp server [35]. Computational analysis of the protein's internal surface areas was performed using the CASTp web server [36], which aided in the identification and characterization of the most prominent residues in the binding pockets.

### 2.7 Ligand Preparation for Docking Analysis

PubChem database was utilized (https://pubchem.ncbi.nlm.nih.gov/) to identify potential AChE inhibitors among phytochemicals derived from *X. strumarium*. A total of 12 different chemicals were found. Using the Chem-Draw Ultra program (version 12.0.2.1076, 2010), the chemical structures of these compounds were saved in the “mol” format. By using Discovery Studio (Biovia 2017), chemical structures in the .mol format were translated to the PDB format. Next, ligand structures were examined using ADT with respect to additions of Gasteiger modifications, rotatable bonds, and combinations with nonpolar hydrogens and components of the ligand. The ligand was then transferred to PDB format. ADT-enabled PDBQT format was employed for AutoDock4 (AD4) and AutoDock Vina [37].

### 2.8 Docking Methodology

The AutoDock Vina programme was utilised to carry out molecular docking. With grid coordinates (grid centre) and specific-sized grid boxes for every receptor, ligands were docked one at a time to the receptor. When the ligand interacted with rigid macromolecules, it was in a flexible state. To launch AutoDock Vina, Notepad was opened, and the configuration file was uploaded. The octopamine input (.PDBQT) file was prepared, and ADT was also needed to configure the size and centre of the grid box. The octopamine structure had polar hydrogen atoms and Kollman charges. The grid centre was assigned at x, y, and z dimensions of 108.914, 105.342, and 147.243, respectively, with a grid spacing of 1000 Å. The grid size was set at 20 × 27 × 20 (x, y, and z) points. The file that was ready was saved in the PDBQT extension. Based on the AutoDock Vina scoring system, negative Gibbs free energy (∆G) scores (kcal/mol) were predicted to indicate ligand-binding affinities [37]. PyMOL and Discovery Studio Biovia 2017 were used to visualise post-docking analyses, which showed the locations and sizes of binding sites as well as hydrogen-bond and hydrophobic interactions. The octopamine active site was coupled to compounds. Following the observation of each ligand's binding positions and the characterization of their interactions with the protein, the most effective compound was determined.

### 2.9 ADMET Evaluation of the most Promising Leads

Early assessment of therapeutic chemical fates in the biological system is crucial to modern drug discovery and development because it helps separate non-drug-like candidates from the pool of bioactive molecules. Absorption (A), distribution (D), metabolism (M), excretion (E), and toxicity (T) of possible drug candidates in the biological system dictate their fate. SwissADME (www.swissadme.ch/) and pkCSM were used to assist in the ADMET evaluation of the chosen bioactive compounds.

### 2.10 Molecular Dynamics Simulation

Protein bioactivities are influenced by their structural dynamics. However, studying protein flexibility through wet lab research is frequently too difficult or unfeasible, requiring the use of *in silico* methods. By merging coarse-grained simulation models with the reconstruction of predicted structures to all-atom representation, it is possible to computationally affordably investigate protein flexibility in biological systems. The CABSflex 2.0 server, available at http://biocomp.chem.uw.edu.pl/CABSflex2, was utilized in order to perform molecular dynamics (MD) simulations of the complex between the *Rmoctopamine* receptor and the optimal ligand (Xanthatin). The protein flexibility of the highest-ranked ligand-protein complexes was assessed using the CABS-flex 2.0 server (http://biocomp.chem.uw.edu.pl/CABSflex2), and the results were displayed using root mean square fluctuation, or RMSF. With significantly lower system requirements, CABS-flex provides dynamic protein simulation and offers quick protein flexibility simulation. According to reports, the flexibility simulation from this server and the NMR data have a strong correlation [38, 39]. As CABS-flex offers high-resolution (10-ns) protein near-native protein dynamics simulation, it is a very useful tool for real-time protein-ligand stability assessment. The CABS-flex simulation was run using 50 cycles with the default parameters.

### 2.11 Statistical Analysis

For all statistical studies, R and RStudio were used. The information was organized using Microsoft Excel (v 2302).) prior to being loaded into the R computer environment for additional statistical analysis. R was used to generate descriptive statistics, such as standard deviation ± mean. A one-way analysis of variance (ANOVA) was used with Post Hoc Tukey’s honestly significant difference (HSD) test to determine the significance of the difference between various concentrations. Furthermore, 50%, 90%, and 99% lethal concentrations (LC_50_, LC_90_, and LC_99_) were determined using the R “ecotox” package. To visualize the data, the R packages “ggplot2 and ggpubr” were used.

## 3 RESULTS

### 3.1 *In vitro* Bioassay

The larvicidal and ovicidal activities of *X. strumarium* extract against *R. microplus* were evaluated at different concentrations. The results of the larval packet test (LPT) and adult immersion test (AIT) are presented in Table **1** and Fig. (**1**).

In the LPT, the highest mortality rate (92 ± 2.646) was observed at the 40 mg/mL concentration of *X. strumarium* extract, which was comparable to the positive control group (92 ± 3.606). As the concentration of the extract decreased, a significant reduction in larval mortality was observed, with the lowest mortality rate (51 ± 2) recorded at the 2.5 mg/mL concentration.

Similarly, in the AIT, the highest egg inhibition (77.057 ± 2.186) was observed at the 40 mg/mL concentration of the extract. A significant decrease in egg inhibition was noted as the concentration of the extract reduced, with the lowest egg inhibition (11.441 ± 5.405) recorded at the 2.5 mg/mL concentration.

The lethal concentrations (LC_50_, LC_90_, and LC_99_) of the *X. strumarium* extract were calculated and are presented in Table **2**. The LC_50_, LC_90_, and LC_99_ values were found to be 2.253 mg/mL, 29.417 mg/mL, and 238.934 mg/mL, respectively. The slope, intercept, and standard error values were consistent across all lethal concentrations.

These results indicated that the *X. strumarium* extract exhibited significant larvicidal and ovicidal activities against *R. microplus*, with the effectiveness of the extract increasing with its concentration. However, further studies are needed to understand the mechanism of action of the extract and to evaluate its potential for use in tick control programs.

The complete sequence of the *R. microplus* octopamine protein was obtained from the UniProt database (Accession No. A7TZ09_RHIMP) as its 3D structures were not available in the PDB [40]. Utilizing the BLASTp algorithm, templates closely resembling the query sequence were identified from the PDB. Two homology modeling tools, Phyre2 and Swiss server, were employed to generate 3D models, subsequently refined through energy minimization techniques using Swiss-SPD Viewer and the GROMOS96 force field [41]. The template c6k41R, with 57% sequence identity and a resolution of 1.75 Å, was selected as the optimal option for constructing the 3D model of the *R. microplus* octopamine protein. Additionally, the Swiss Model employed template 6kux.1.A, with a sequence identity of 38.8%, for model construction.

### 3.2 Examination and Verification of the Simulated Structures

**Fig. (2):**
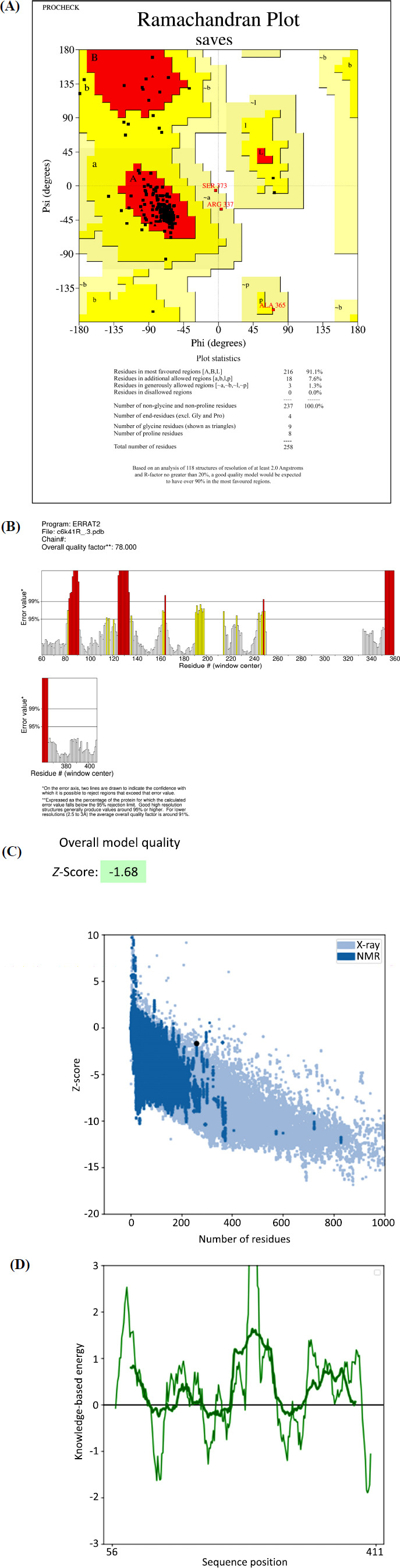
*R. microplus* octopamine three-dimensional (3D) structure predicted by the Phyre 2 server; (**A-D**) Ramachandran map, ERRAT, Z-scores, and, respectively, validate the protein structure and amino acid position of RmGST. The Ramachandran plot of Rm octopamine indicates the percentage of residues in favored regions (red) and allowed regions (yellow), where the bars in the ERRAT plot represent the error value (white: error < 95%, yellow: error < 99%, and red: error > 99%).

The structural evaluation post-optimization of the 3D model was determined using the Ramachandran map (PROCHECK). The u and w distribution of the Ramachandran diagram, which is produced by non-glycine, non-proline residues, was determined using the PROSA web online server (Tables **3** and **4**). The psi and pi distributions of the Ramachandran Map, along with the amino acids of the protein in the preferred region, indicated that the projected models of the *R. microplus* octopamine receptor were well-constructed and highly reliable. The phi and psi angles were plotted against each other to show which locations were favorable and which were not. We evaluated the quality of various regions using these parameters. Findings from the PROCHECK analysis of Phyre 2 showed that amino acid residues were distributed as follows: 7.7% of residues were in allowed regions, 0.9% were in the generous region, and 0.3% were in prohibited areas; 91.1% of residues were in preferred areas, while for the Swiss model, 7.6% were in allowed regions, 1.3% were in the generous region, and 0.0% were in prohibited areas. The PROSA web server, based on the Z-score, was used to contrast the modeled protein structure with existing protein structures sourced from PDB [42]. The software presented the input structure's Z-score and the plot of residue energies. The Z-score for Phyre2 had Z-scores of -1.68 while the Swiss model was -3.3, respectively, suggesting that the overall model quality of the Swiss model was satisfactory (Fig. **2**). For the Phyre 2 model, ERRAT produced a quality factor of 78.000%, but the Swiss model has a quality factor of 92.420%. The degree of certainty with which the regions that exceeded the error value may be rejected was represented by two lines on the error axis. The fraction of the protein for which the calculated error value is smaller than the 95% rejection threshold is displayed. Through a comparison between statistical analysis and greatly enhanced predicted structures, ERRAT yielded the error function value and confidence bounds. As a result, the total value of both models was considered significant. It was concluded that the Phyre2 was more suitable than the Swiss model and was, therefore, selected for subsequent studies.

### 3.3 Docking Analysis

**Fig. (3):**
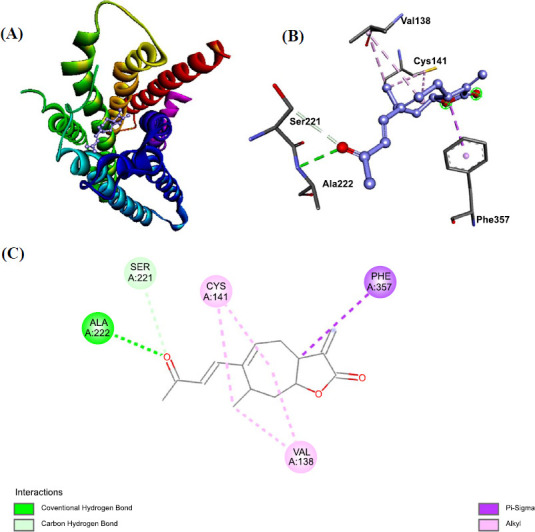
(**A**-**C**) Depicting 2D and 3D bondings of Xanthatin with octopamine receptor of *R. microplus*.

**Fig. (4):**
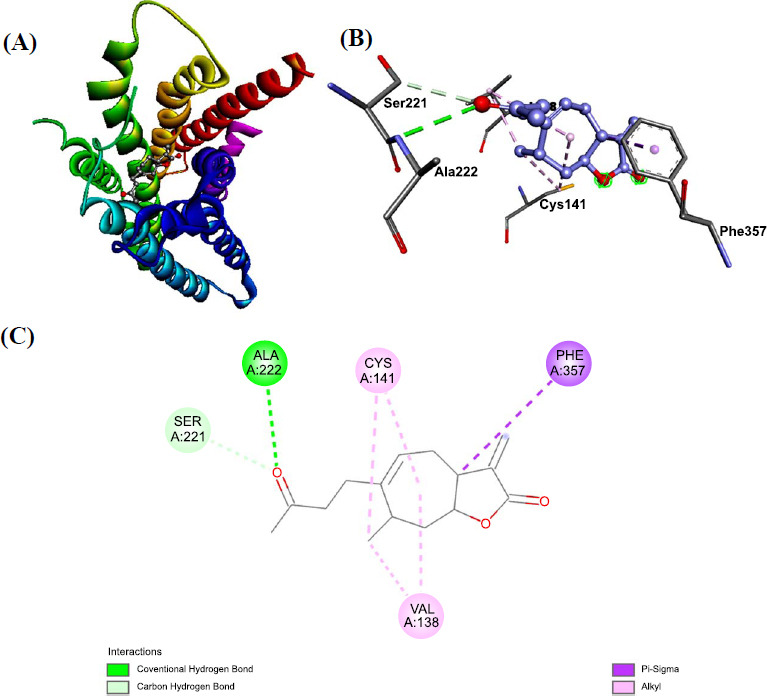
(**A**-**C**) Displaying 2D and 3D bondings of Xanthosin with octopamine receptor of *R. microplus*.

When potential ligands were docked with *R. microplus* octopamine using ADT, the results indicated that Xanthosin and Xanthatin had the lowest (1^st^) conformation and a ∆G score of -9.4 and -9.2 kcal/mol, respectively. According to these findings, Xanthatin and Xanthosin were found to have the highest binding affinity for the target protein among the twelve ligands. Using AutoDock Vina, we conducted simultaneous studies to map the Octopamine amino acid residues interacting with the ligands through hydrogen bond, hydrophobic, and electrostatic interactions (Figs. **3** and **4**). Phenol 138 (2.89), Cys 141 (2.75), Phe 357 (4.25), and Ala222 (2.89) amino acid residues were the sites of three hydrophobic contacts that xanthatin created. Additionally, Ser221 (3.24) and the target receptor produced one carbon-hydrogen bond. Moreover, *R. microplus* octopamine was found to be reacted with Xanthosin.

### 3.4 Drug-likeness and ADMET Analysis

Molecular mass (less than 500 daltons), hydrogen bond donors/HBD (less than 5), hydrogen bond acceptors/HBA (less than 10), octanol/water partition coefficient/log p (less than 5), and molecular refractivity (40-130) are the five primary parameters. The drug-likeness analysis of this study was conducted in accordance with Lipinski's Rule of Five (Ro5) [43, 44]. Table **3** indicates that all of these ligands, with the exception of chlorogenic acid, which has a hydrogen bond donor of 5, have molecular weights less than 500 daltons. The hydrogen bond acceptors of these ligands are less than 10 in each case. The surface area of ligands ranges from 1.368 to 201.354, while their logP values range from -0.6459 to 8.1689.

Skin permeability, intestinal absorption, and water solubility characteristics make up absorption prediction. According to the table, the water solubility of the compounds ranged from -1.668 to -3.127 log mol/L. Less than 0.5 is the optimum value for water solubility, and less than 0 is the best category value, indicating that these substances have good water solubility in the body [45]. With skin permeability ranging from -1.721 to -4.289 log Kp, respectively, these three compounds are also classified as non-sensitizers.

The blood-brain barrier (BBB) and volume distribution (VDss) were included in the drug distribution parameters. According to the table, all of these compounds, aside from four, were good for drug distribution in the blood, with good VDss falling between 0.5 and 3 L/Kg [46]. The criteria used to assess drug distribution using BBB and CNS permeability characteristics are high absorption (>2.0), moderate absorption (0.1-2.0), and low absorption (<0.1) [47]. Compounds, such as betulinic acid, chlorogenic acid, lupeolacetate, norxanthantolide A, and xanthiazone, had low absorption of log BB, while all other compounds had moderate absorption of log BB. Cytochrome 450 (CYP) inhibition is another factor that affects drug metabolism prediction [48]. With the exception of lupeolacetate and betulinic acid, which inhibit CYP3A4 and CYP2D6, compounds do not inhibit any of the enzymes of CYP1A2, CYP2C19, CYP2C9, CYP2D6, or CYP3A4. It is predicted that these compounds would not have any effect on the digestive system.

Additionally, the total clearance is used to determine the excretion parameter. According to Table **4**, some compounds excrete molecules at the fastest rate (around 1.393 log ml/min/kg), which indicates that they have a greater effect than other compounds. The body excretes drugs more quickly in response to greater overall clearance values [49]. The final pharmacokinetic property measure was acute oral toxicity, as demonstrated by the lethal dosage 50 (LD_50_), a statistical metric that determines the number of animals who die in 50% of cases when multiple medications are given as a single dose at a particular time [50, 51]. Table **4** indicates that compounds might be dangerous to consume with an LD_50_ ranging from 1.88 to 2.478 mol/kg, respectively. All compounds showed no hepatotoxicity except betulinic acid and xanthosin.

### 3.5 Molecular Dynamic Simulation

**Fig. (5):**
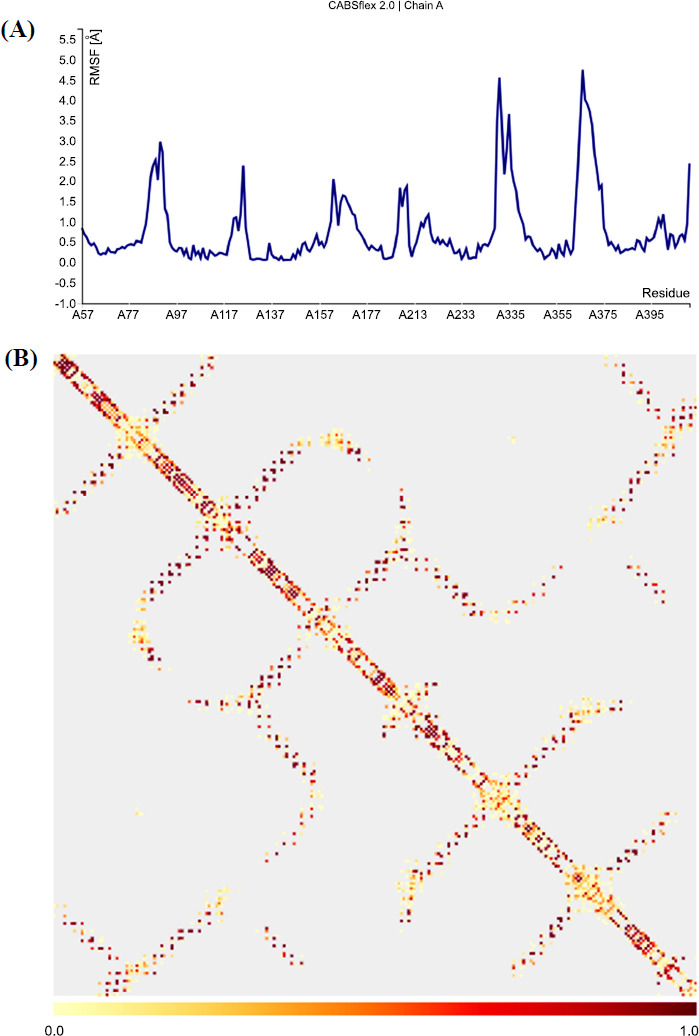
(**A**) Fluctuation plot of *R. microplus octopamine*. (**B**) Contact map of the residues of *R. microplus octopamine*.

To determine the stability of the interaction between ligand 3 and the target protein, an MD simulation analysis of the protein-ligand complex was performed using the CABS-flex 2.0 server (http://biocomp.chem.uw.edu.pl/CABSflex2). Most of the active residues of the target macromolecule exhibited consistent fluctuations within the range of less than 2.0 Å (Fig. **5**). This implies that the protein structure is stable and does not substantially change from what it was originally structured. Residues A335 (4.3 Å) and A368 (4.8 Å) showed a notable divergence that may be the consequence of structural modifications the protein experiences. The different visualizations in the model provide evidence of the macromolecule's structural heterogeneity. Additionally, the “contact map” provides an extensive perspective of the protein's residue-residue interaction pattern (Fig. **5B**). The hue of the map indicates how frequently these interactions occur. Furthermore, every dot on the map depicts an interaction between two residues. The presence of deep dark hues on a scale of 1.0 indicates that there are strong interactions between the residues of the *R. microplus* octopamine protein target (Fig. **5**).

## 4 DISCUSSION

*R. microplus*, widely referred to as the cattle tick, is a prominent ectoparasite impacting cattle throughout the globe. The overuse of the chemical acaricides has resulted in tick resistance, providing a severe challenge for the cattle industry. Consequently, it is essential to discover alternate techniques to reduce *R. microplus* infestations.

In this research, we studied the acaricidal activity of the locally selected plant *X. strumarium* against *R. microplus* by computational analysis, followed by *in vitro* experiments. The experiment involved testing ticks with various concentrations of methanol extract (2.5, 5, 10, 20, and 40 mg/mL). The results indicated that at a dosage of 40 mg/mL, the plant extracts from *X. strumarium* exhibited the highest mortality rates, reaching 92%. The aim was to identify novel herbal acaricides and their phytoconstituents for effective tick control. A significant number of plant species have demonstrated acaricidal characteristics, with the majority of the examined plants belonging to diverse botanical families [52, 53].

Numerous botanical species have been the subject of previous investigations regarding their potential acaricidal properties against Culicoides [54], ticks [55, 56], and mites [57, 58]. The successful large-scale production of essential oils has facilitated the profitable utilization of various plant species, which exhibit both repellent and anti-parasitic properties, against a diverse range of parasites [59].

*X. strumarium* possesses various therapeutic properties, including cooling, laxative, fattening, anthelmintic, alexiteric, tonic, digestive, antibacterial, and antipyretic effects [60]. Additionally, *X. strumarium* has been documented as a traditional remedy for the management of urinary disorders, ear infections, diabetes, and gastric disorders and also for the treatment of leucoderma, insect bites, epilepsy, and biliousness [61, 62].

*X. strumarium* contains various phenolic compounds, triterpenoid saponins, and xanthanolide sesquiterpene lactones [63]. The two main and most prevalent bioactive substances in *X. strumarium* are sesquiterpenes and phenylpropanoids, which are regarded as distinctive elements of this plant. Notably, sesquiterpenoids, such as 2-hydroxy xanthinosin, xanthnon, xanthatin, xanthinosin, isoxanthanol, xanthumin, and xanthinin, have been identified in the leaves [60]. An essential step in identifying novel drugs is utilizing a validated target to screen biochemical libraries *in vitro*. *In vitro* screening has benefited the development of treatments for cancer, cardiovascular and kidney disease, as well as infection control for various disease-causing agents (viruses, bacteria, parasites, and fungi) [64]. One factor to consider when selecting a molecular target is selectivity. A pathogen's chosen target needs to be different from the biomolecules of the host in order to be targeted. *In silico* experiments were carried out to gain more insight into the interactions between the phytochemicals present in *X. strumarium* and the *R. microplus* Octopamine receptor.

In this research, a docking study was performed through the AutoDock Vina program. The best model found through homology modelling was used for the docking simulation. Twelve phytochemical compounds were also employed in the docking research as ligands. The findings from the docking study were analyzed to identify the best binding modes among various combinations. To identify potential acetylcholinesterase inhibitors, a library of phytochemicals from *X. strumarium* interacted with different binding sites of an octopamine receptor using the AudoDock vina software. Among the tested compounds, xanthathin from *X. strumarium* exhibited the highest inhibitory activity, with a docking score of -9.4 kcal/mol. This compound demonstrated unique interactions with the amino acids of the octopamine receptor, contributing to its effectiveness as an inhibitor. Xanthosin from *X. strumarium* was identified as the second most potent inhibitor, with a docking score of -9.2 kcal/mol.

Due to their acaricidal and repellent effects on ticks, natural plant-derived chemicals have gained significant interest as tools for integrated pest management programs. The industry's interest, along with the proven efficacy of various chemicals and their reduced risks to humans and the environment, highlights the importance of research in this field. Scientific advancements have led to the development of several commercial products based on plant bioactive chemicals. In summary, understanding the acaricidal properties of plants present in local and regional environments is crucial for the development of safe, effective, affordable, accessible, ecologically sound, and community-driven tick control programs.

## CONCLUSION

In conclusion, this research highlights the potential of the ethanolic extract of *X. strumarium* to exhibit acaricidal properties against *R. microplus* ticks. *X. strumarium* has considerable potential as a natural treatment option for tick control due to its dose-dependent death rates and improved effectiveness compared to permethrin. Molecular docking analysis identified xanthatin and xanthosin as possible drug candidates with high binding affinities to target proteins, providing valuable information on the biochemical pathways that contribute to their anti-tick activities. The evaluation of physicochemical qualities and ADME characteristics provides further evidence for the suitability of these phytochemicals as possible non-toxic treatments for combating ticks. These compounds possess advantageous drug-like characteristics and solubility profiles, making them a promising basis for the advancement of tick control strategies that are ecologically sustainable. The present study makes a significant contribution to the domain of tick management by shedding light on the potential of *X. strumarium* as a viable and enduring tick control agent.

## Figures and Tables

**Fig. (1):**
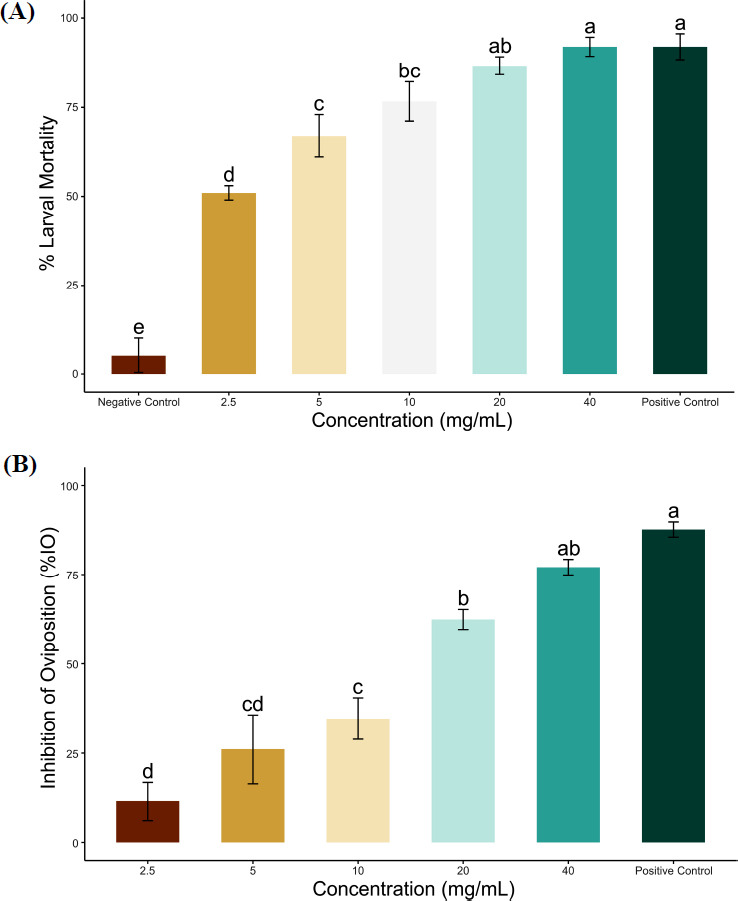
The mortality at various concentrations for (**A**) larval packet test and (**B**) adult immersion test, as well as the significant difference between mortalities at various concentrations of the extract.

**Table 1:** Mean ± standard deviation of the larval mortalities of *R. microplus* treated with *X. strumarium* extract in LPT and egg inhibition in the AIT.

Group	Concentration	Replicates	LPT	AIT
			**Mean ± SD**	**Mean ± SD**
*X. strumarium*	40	3	92 ± 2.646^a^	77.057 ± 2.186^ab^
	20	3	86.667 ± 2.517^ab^	62.413 ± 2.79^b^
	10	3	76.667 ± 5.508^bc^	34.666 ± 5.795^c^
	5	3	67 ± 6^c^	26.07 ± 9.609^cd^
	2.5	3	51 ± 2^d^	11.441 ± 5.405^d^
Control Group	Positive	3	92 ± 3.606^a^	87.535 ± 2.13^a^
	Distilled Water	3	5.333 ± 4.933^e^	-54.647 ± 10.185^e^

**Note**: SD: Standard deviation, a-e: the superscripted same letters in a column represent the significance difference with a *p*-value of 0.05 or less (95% confidence) according to the Post Hoc Tukey’s Honestly Significance Difference.

**Table 2:** The lethal concentrations at which 50% (LC_50_), 90% (LC_90_), and 99% (LC_99_) of the test tick populations can be killed *in vitro* at 24 h.

**LC**	**n**	**Concentration (mg/mL)**	**LCL**	**UCL**	**Slope ± SE**	**Slope *p*-value**	**Intercept ± SE**	**Intercept *p*-value**	**SE**	**χ^2^**	**df**	***p*-value**	**h**	**z**	**var_m**	**Covariance**
LC_50_	15	2.253	1.707	2.796	1.149 ± 0.091	0	-0.405 ± 0.089	0	1.131	10.639	13	0.641	1	12.557	0.03	-0.007
LC_90_	15	29.417	23.49	39.367	1.149 ± 0.091	0	-0.405 ± 0.089	0	1.138	10.639	13	0.641	1	12.557	0.002	-0.007
LC_99_	15	238.934	147.636	458.785	1.149 ± 0.091	0	-0.405 ± 0.089	0	1.326	10.639	13	0.641	1	12.557	0.004	-0.007

**Abbreviations**: LC: lethal concentration; n: number of variables; LCL: lower confidence limits; UCL: upper confidence limits; SE: standard error; χ^2^: chi-square; df: degree of freedom; h: null hypothesis.

**Table 3:** Druglikeness and molecule properties of *X. strumarium* compounds through the Swiss ADMET tool.

**Compounds**	**Molecular Weight (g/mol)**	**LogP**	**Rotatable Bonds**	**Acceptors**	**Donors**	**Surface Area**
Limonene	136.23	2.72	1	0	0	1.368
8-epi-xanthatin	246.306	2.5857	2	3	0	107.205
Betulinic acid	456.711	7.0895	2	2	2	201.354
Chlorogenic acid	354.311	-0.6459	4	8	6	141.587
Lasidiol	238.371	2.8908	1	2	2	104.736
Norxanthantolide A	194.23	1.3292	0	3	0	83.124
Xanthatin	246.306	2.5857	2	3	0	107.205
Xanthiazone	239.296	0.5886	1	4	2	97.788
Xanthinin	306.358	2.3513	4	5	0	129.899
Xantholide B	232.323	3.0965	0	2	0	102.727
Xanthosin	284.228	-2.9756	2	8	5	109.829
β-amyrin	426.729	8.1689	0	1	1	192.398

**Table 4:** Pharmacokinetic profile and toxicity prediction of *X. strumarium* compounds through pkCSM webserver.

**Compound**	**Absorption (Water Solubility)**	**Skin Permeability**	**Distribution VDss (Human)**	**BBB Permeability**	**Metabolism CYP2D6 Substrate**	**CYP3A4 Substrate**	**CYP2D6 Inhibitor**	**Excretion Total Clearance**	**Renal OCT2 Substrate**	**Toxicity Oral Rat Acute Toxicity (LD_50_)**	**Hepatotoxicity**
Limonene	-3.568	-1.721	0.396	0.732	No	No	No	0.213	No	1.88	No
8-epi-xanthatin	-3.057	-2.756	0.11	0.294	No	No	No	0.615	No	1.814	No
Betulinic acid	-3.122	-2.735	-1.18	-0.322	No	Yes	No	0.116	No	2.256	Yes
Chlorogenic acid	-2.449	-2.735	0.581	-1.407	No	No	No	0.307	No	1.973	No
Lasidiol	-3.313	-3.094	0.225	0.088	No	No	No	1.129	No	1.949	No
Lupeolacetate	-3.892	-2.671	0.886	-0.039	No	Yes	Yes	1.285	No	2.443	No
Norxanthantolide A	-1.668	-3.146	-0.005	-0.201	No	No	No	0.202	No	1.994	No
Xanthatin	-3.057	-2.756	0.11	0.294	No	No	No	0.615	No	1.814	No
Xanthiazone	-2.038	-4.289	-0.071	-0.236	No	No	No	0.331	No	2.237	No
Xanthinin	-3.048	-3.124	-0.105	-0.392	No	No	No	1.393	No	2.255	No
Xantholide B	-3.827	-2.484	0.42	0.583	No	No	No	0.2	No	1.673	No
Xanthosin	-2.399	-2.735	-0.02	-1.251	No	No	No	0.594	No	1.859	Yes
β-amyrin	-6.531	-2.811	0.268	0.667	No	Yes	No	-0.044	No	2.478	No

## Data Availability

The authors confirm that the data supporting the findings of this research are available within the article.

## LIST OF ABBREVIATIONS

ADMET: Absorption, Distribution, Metabolism, Excretion, Toxicity
AIT: Adult Immersion Test
ANOVA: Analysis of Variance
IE: Index of Egg Laying
IO: Inhibition of Oviposition
LC: Lethal Concentrations
LPT: Larval Packet Test
LT: Lethal Time
OCs: Organochlorides
Ops: Organophosphates
R. microplus: Rhipicephalus microplus
SPs: Synthetic Pyrethroids
X. strumariam: Xanthium strumarium
